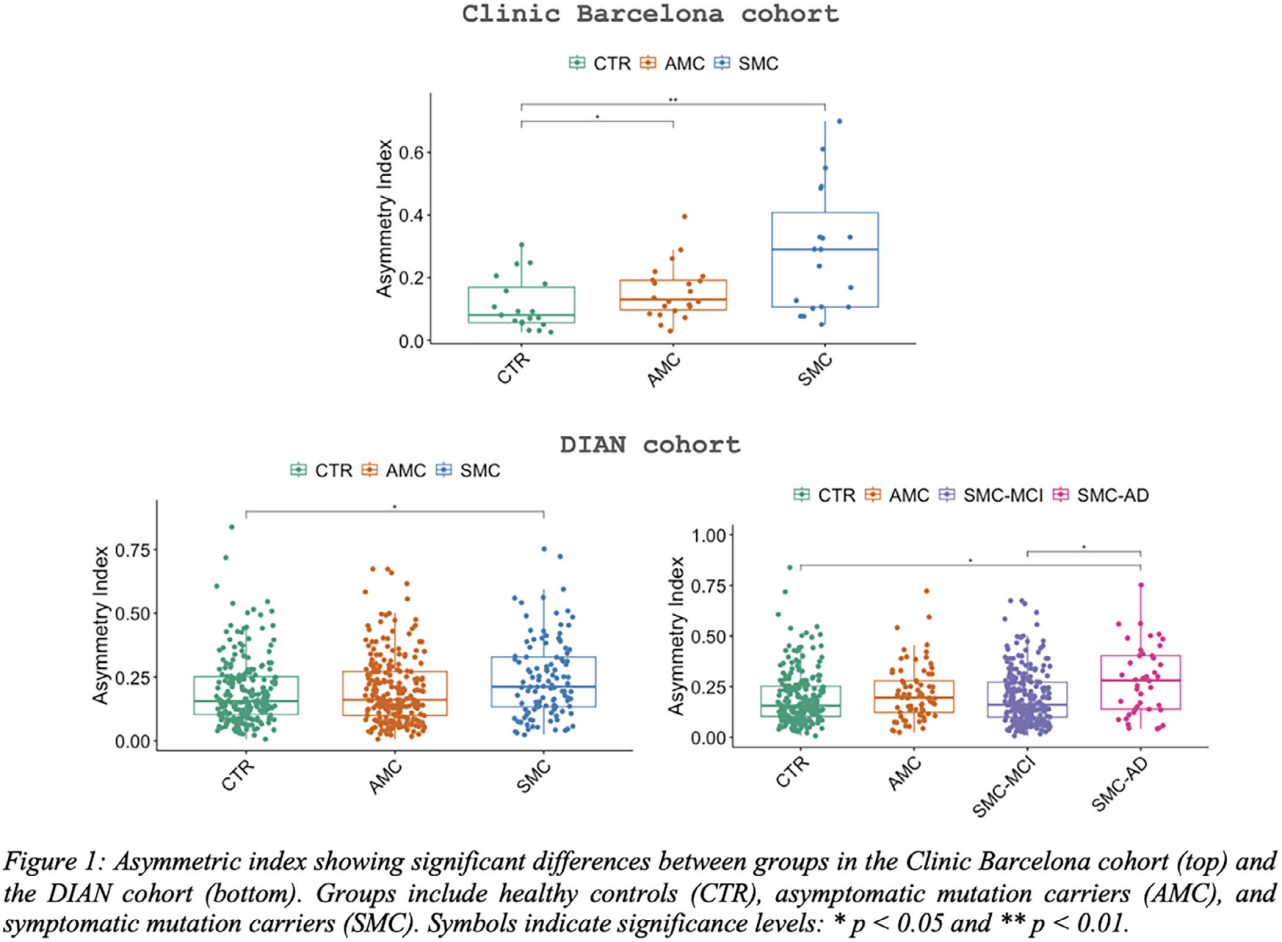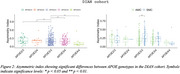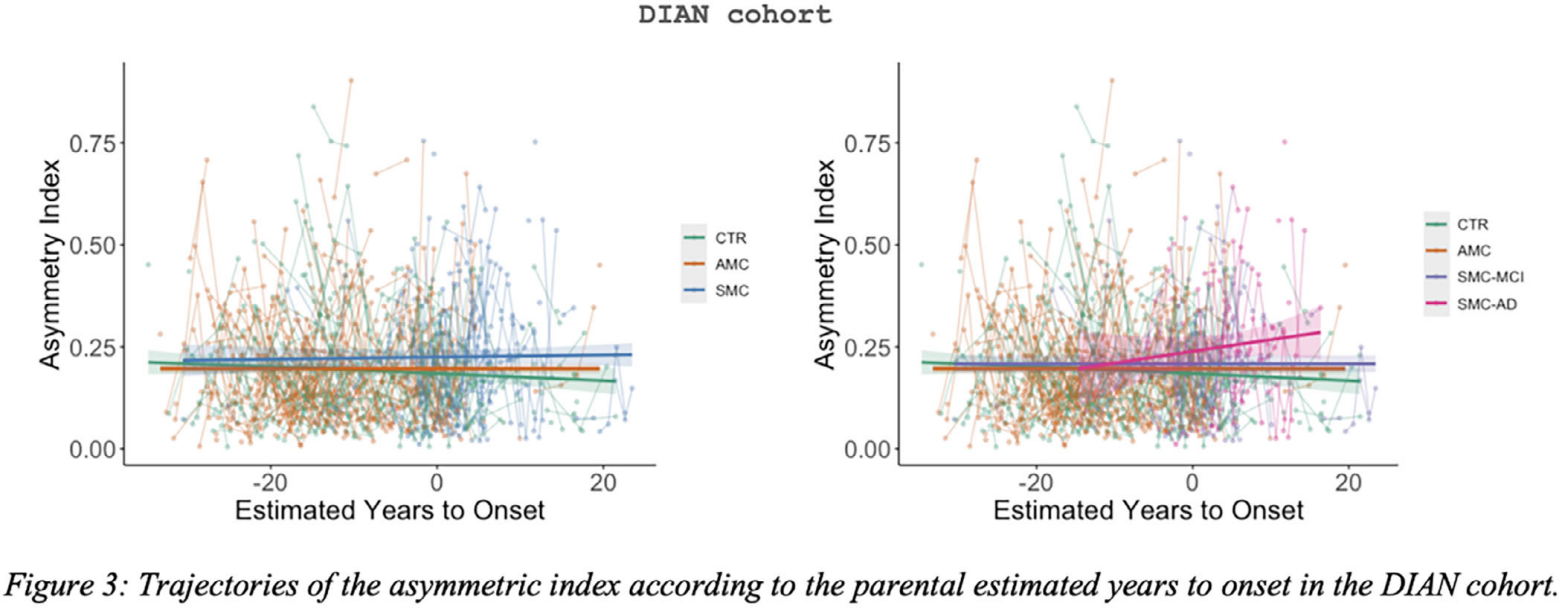# Cortical asymmetry index in Autosomal Dominant Alzheimer’s Disease: insights from the Clínic Barcelona and DIAN cohorts over time

**DOI:** 10.1002/alz70862_110715

**Published:** 2025-12-23

**Authors:** Agnès Pérez‐Millan, Beatriz Bosch‐Capdevila, Sergi Borrego‐Écija, Anna Antonell, Núria Guillén, Guadalupe Fernandez‐Villullas, Adrià Tort‐Merino, Mircea Balasa, Albert Lladó, Neus Falgàs Martínez, Raquel Sánchez‐Valle

**Affiliations:** ^1^ Alzheimer’s disease and other cognitive disorders Group. Service of Neurology, Hospital Clínic de Barcelona. Fundació Recerca Clínic Barcelona‐IDIBAPS, Barcelona Spain; ^2^ eHealth Center, Faculty of Computer Science, Multimedia and Telecommunications, Universitat Oberta de Catalunya, Barcelona Spain; ^3^ Alzheimer's Disease and Other Cognitive Disorders Unit, Neurology Department, Hospital Clinic, Barcelona Spain; ^4^ Alzheimer’s disease and other cognitive disorders Unit. Hospital Clínic de Barcelona. Fundació de Recerca Clínic Barcelona – IDIBAPS. University of Barcelona, Barcelona Spain

## Abstract

**Background:**

The Cortical Asymmetry Index (CAI) evaluates brain asymmetry, showing increased asymmetry in sporadic Alzheimer’s Disease (AD). We investigate CAI in asymptomatic (AMC) and symptomatic (SMC) mutation carriers of Autosomal Dominant Alzheimer’s Disease (ADAD).

**Method:**

Baseline T1‐weighted MRI were collected from the ADAD cohort at Clínic Barcelona (Clinic Barcelona cohort), including SMC (*N* = 19), AMC (*N* = 22), and healthy controls (CTR) (*N* = 19). The Dominantly Inherited Alzheimer Network observational study (DIAN‐OBS) provided longitudinal MRI data as a second cohort (DIAN cohort), including SMC (*N* = 115), AMC (*N* = 234), and CTR (*N* = 215). In the DIAN cohort, SMC participants were subdivided into mild cognitive impairment (SMC‐MCI, CDR=0.5) and AD (SMC‐AD, CDR≥1). Available cerebrospinal fluid (CSF) and plasma neurofilament‐light chain (NfL) levels were included. Cortical thickness was analysed using Freesurfer, and CAI was calculated using the open‐source pipeline. Cross‐sectional analyses assessed diagnosis and *APOE4* differences, adjusting for age, sex, and estimated years from onset (EYO), and correlations between CAI and age, EYO, MMSE, and NfL. Longitudinal progression differences were examined using GAM models according to EYO with the DIAN cohort, using as fixed effects age, sex and the interaction between the group and EYO.

**Result:**

CAI distinguished AMC and SMC from CTR in Clinic Barcelona cohort, and differentiated SMC‐AD from CTR and AMC in DIAN cohort (Figure 1). Carriers and SMC in the Clinic Barcelona cohort exhibited higher CAI, linked to elevated plasma‐NfL, advanced EYO and lower MMSE. In DIAN cohort, carriers showed higher CAI associated with elevated NfL (plasma and CSF), reduced MMSE and advanced EYO (also for SMC‐AD). DIAN cohort *APOE3/3* showed differences from other *APOE* genotypes in carriers and distinctions between AMC and SMC (Figure 2). In the DIAN cohort, SMC and SMC‐AD presented a significant CAI increase over time (Figure 3).

**Conclusion:**

ADAD individuals show increased brain asymmetry as the disease progresses and correlate with key biomarkers in both cohorts. *APOE3/3* showed higher levels of asymmetry than the other *APOE* genotypes. Longitudinally, CAI increases significantly in SMC and SMC‐AD, highlighting its potential as a marker for disease monitoring. These findings highlight CAI's potential as a tool for early detection and tracking of AD progression